# Long-Term Outcomes Following Nonoperative Treatment of Prearthritic or Extra-Articular Hip Pain in Women

**DOI:** 10.1089/whr.2022.0022

**Published:** 2022-08-04

**Authors:** Campbell Goldsmith, Jennifer Cheng, Joanna Halpert, Peter Moley

**Affiliations:** Department of Physiatry, Hospital for Special Surgery, New York, New York, USA.

**Keywords:** nonoperative, prearthritic hip pain, women, outcomes

## Abstract

**Introduction:**

There is an abundance of literature focusing on morphological and surgical outcomes in women with arthritic and prearthritic hip pain. However, no studies have evaluated conservative treatment outcomes, such as physical therapy (PT) and injections, in women with prearthritic or extra-articular hip pain. The purpose of this study is to assess changes in long-term patient-reported outcome measures after nonoperative treatments in women with prearthritic or extra-articular hip pain.

**Methods:**

Twenty-nine female patients (35–65 years old) who presented to a single provider between December 1, 2012 and September 1, 2017 for prearthritic or extra-articular hip pain (Tonnis 1 or less) and had baseline patient-reported outcome data (modified Harris Hip Score [mHHS], Hip Outcome Score [HOS] activities of daily living [ADL] and sport scores, International Hip Outcome Tool-33 [iHOT-33]) available from the institutional hip registry were included. Patients underwent nonoperative treatments for intra-articular or extra-articular hip pain. A follow-up questionnaire was prospectively administered at 3–5 years after the baseline visit.

**Results:**

Most patients underwent targeted PT (*n* = 27; 93%) to treat intra-articular or extra-articular hip pain. Targeted PT can be defined as primarily exercise-based therapy focusing on hip and lumbar stability. Twelve patients (41%) received injections; of these, 11 were also treated with PT. Overall, significant improvements in mHHS, HOS-ADL, and iHOT-33 scores were observed (*p* = 0.006, 0.022, and <0.001, respectively). HOS-ADL and iHOT-33 scores improved by a median of 10.3 and 18.0 points, respectively, and were clinically significant. HOS-sport scores also improved but were not statistically significant. There were no differences in patient-reported outcomes between patients who received both PT and injections versus those who received PT, injections, or other treatments.

**Conclusions:**

Nonoperative treatments for prearthritic or extra-articular hip pain in women, specifically PT and/or injections, were associated with sustained improvements in patient-reported outcomes at 3–5 years postbaseline.

## Introduction

Generalized hip pain is commonly attributed to varying conditions, including osteoarthritis (OA), femoroacetabular impingement (FAI), hip dysplasia, femoral version, tendinitis of the hip joint, degeneration of the labrum, and chondral lesions.^1–3^ Chronic hip joint pain, also known as intra-articular hip disease and prearthritic hip disease, severely limits patient activity. In recent years, the number of studies investigating the prearthritic hip has increased, specifically observing treatment mechanisms to avoid the development of OA requiring surgical intervention.^4–7^ Common treatment options include physical therapy (PT), medication, activity modification, patient education, ultrasound/fluoroscopic-guided therapeutic injections, and surgery.^8^

Prearthritic hip pain that is left untreated has the potential to progress to early OA, highlighting the importance of understanding the mechanisms of injury and pain in these patients. In young adults between 18 and 30 years old, differences in hip morphology between men and women have been observed, such that women have smaller alpha angles and increased anteversion.^9^ Joseph et al. observed patients undergoing arthroscopic surgery for FAI and showed that women had poorer self-reported hip function preoperatively. No differences in hip function were found between men and women at 2 years postoperatively.^10^

In addition, Malviya et al. observed that women reported lower quality of life than men both before and after hip arthroscopy for the treatment of FAI.^11^ Furthermore, Salvo et al. demonstrated that men and women undergoing hip arthroscopy differed in preoperative hip function, morphology, and self-reported functional deficits. However, men and women did not differ on symptom location, duration, or onset.^12^ Several studies have supported the finding of sex-dependent differences in hip function, morphology, and patient-reported outcomes in patients receiving hip arthroscopy.^9,13,14^ In patients presenting with symptomatic hip labral tears, Lindner et al. observed sex differences in hip structure, biomechanics, and operative findings.^15^ Finally, Meghpara et al. reported that both men and women demonstrate improved outcomes and clinical effectiveness at a minimum of 2 years after gluteus medius repair.^16^

Although it is clear that the literature has evaluated surgical outcomes for prearthritic hip pain in women and sex-based morphological differences, no studies to date have evaluated long-term outcomes specifically for women with prearthritic hip pain after nonoperative treatments, such as PT and injections. Along the same lines as previously cited surgical studies, certain conservative treatment options may work better for women in comparison with men when presenting with prearthritic or extra-articular hip pain. Studying these specific outcomes as it pertains to female patients is an essential first step to treating patients with the highest standard of specialized care, further improving our treatment options and strategies. Thus, this study aims to assess and report on long-term patient-reported outcomes after nonoperative treatments in women presenting with prearthritic or extra-articular hip pain.

## Methods

### Ethics and recruitment

This retrospective study with prospective follow-up was approved by the Institutional Review Board (IRB No. 2017-1098), and verbal informed consent was obtained from all patients. The verbal informed consent process was approved by the Institutional Review Board. Female patients (35–65 years old) who presented to a single provider with prearthritic or extra-articular hip pain, were part of the institutional hip registry, had available baseline patient-reported outcome data from the registry, and completed a follow-up at 3–5 years postbaseline visit were included in the study. Exclusion criteria included male patients, ages <35 or >65 years, and arthritic-related hip pain (Tonnis grade: 2+). The age range of 35–65 years old was selected, as it was the nature of the respective group and an efficient way to rule out an indication of OA.

### Outcomes and data collection

Demographic and treatment data were collected from electronic medical records. The modified Harris Hip Score (mHHS), Hip Outcome Score (HOS; activities of daily living [ADL] and sports scales), and International Hip Outcome Tool-33 (iHOT-33) were administered to patients at their baseline visits, as part of the registry. These outcome measures were also prospectively administered at 3–5 years after the baseline visit, as part of this study. Increases in scores over time represent improvements in outcomes. The minimal clinically important differences (MCIDs) are 8.2, 8.3, 14.5, and 12.1 points for mHHS, HOS ADL, HOS sports, and iHOT-33, respectively.^17^

### Statistical analysis

This was a convenience sample of patients who were part of an institutional registry and completed the prospective follow-up at 3–5 years after their baseline visits. Continuous variables are reported as medians and interquartile ranges (IQRs), whereas discrete variables are reported as frequencies and percentages. The Wilcoxon rank-sum test was used to compare follow-up outcome scores with baseline. Statistical significance was defined as *p* < 0.05. All analyses were performed with Stata, version 14.2 (StataCorp., College Station, TX).

## Results

### Patient flow and baseline information

A total of 29 patients with complete data sets were included in the study. The median age at the time of the baseline visit was 49 years (IQR: 43–53), and 28 (97%) patients were of Caucasian race. The median body mass index was 22.1 kg/m^2^ (IQR: 20.7–26.3). One patient had concurrent knee pain; none of the patients had concurrent back or hamstring pain. The median duration of pain was 6.5 months (IQR: 5.5–13). Diagnoses included gluteal tendinitis or gluteus medius tendinopathy (*n* = 13), hip impingement (*n* = 9), psoas tendinitis (*n* = 9), dysplasia (*n* = 6), osteitis pubis (*n* = 1), and right inferior pubic ramus fracture (*n* = 1).

### Nonoperative treatments

Most patients underwent targeted PT (*n* = 27; 93%) that focused on both lumbar and hip stability to treat their prearthritic or extra-articular hip pain. Patients attended 1–2 sessions of PT per week for a duration of 6 weeks. For targeted PT, therapists were instructed to focus on exercise-based therapy of the hip, specifically focusing on balance training, biomechanics, hip adductor strengthening, low-back strengthening, glut/hip abductor strengthening, posture, body mechanics, trunk stabilization, spine stabilization, core strengthening, and small muscle control. PT was provided in a stepwise progression with functional goals. The standardized prescription used for PT is shown in Appendix Table A1.

Twelve patients (41%) received injections; 2 (16.7%) received hamstring platelet-rich plasma (PRP) injections, 7 (58.3%) received ultrasound-guided corticosteroid injections of the hip joint, 2 (16.7%) received ultrasound-guided trochanteric bursa corticosteroid injections, and 1 (8.3%) received a facet joint injection. Of these 12 patients who received injections, 11 were also treated with PT. Other reported treatments included medications (*e.g.*, nonsteroidal anti-inflammatory drugs, Tylenol), acupuncture, icing, and yoga.

### Outcomes

The mHHS, HOS ADL, HOS sports, and iHOT-33 scores at baseline and at 3–5 years postbaseline visit are shown in Table 1. Significant improvements in mHHS, HOS ADL, and iHOT-33 scores were observed (*p* = 0.006, 0.022, and <0.001, respectively). HOS ADL and iHOT-33 scores improved by a median of 10.3 and 18.0 points, respectively, thus meeting MCID criteria for both outcome measures. HOS sport scores also improved, although statistical significance was not met. One patient underwent hip surgery during the 3- to 5-year follow-up period; all outcomes worsened for this patient. A closer look at the treatments revealed no differences in any of the patient-reported outcomes between patients who received both PT and injections versus those who received PT, injections, or other treatments alone (Table 2).

**Table 1.** Patient-Reported Outcomes in Women

Outcome measure	Baseline (***n*** = 29)	3–5 years postbaseline (***n*** = 29)	Change in scores^a^	***p***
mHHS	70.4 (53.9–79.2)	77 (67.0–87.0)	6.6 (13.1–7.8)	0.006
HOS ADL	82.3 (55.2–89.7)	91.2 (83.3–97.1)	10.3 (26.0–8.8)	0.022
HOS Sport	61.1 (21.9–83.3)	75 (65.6–94.4)	13.1 (43.8–11.1)	0.067
iHOT-33	47.5 (33.5–58.6)	65.5 (54.3–82.8)	18.0 (20.7–24.3)	<0.001

Results are medians (interquartile ranges).

^a^ Parentheses represent change in quantiles.

ADL, activities of daily living; HOS, Hip Outcome Score; iHOT-33, International Hip Outcome Tool-33; mHHS, modified Harris Hip Score.

**Table 2.** Changes in Patient-Reported Outcomes After Physical Therapy + Injection Versus Other Treatments

	Both PT and injection treatments (***n*** = 11)	Other treatments^a^ (***n*** = 18)	***p***
Change in mHHS score	8.1 (5.6 to 19.9)	2.4 (−2.9 to 14.3)	0.252
Change in HOS ADL score	7.2 (−10.4 to 14.3)	5.6 (0.0 to 27.5)	0.603
Change in HOS Sport score	12.9 (−15.2 to 24.0)	4.3 (−6.9 to 20.7)	0.610
Change in iHOT-33 score	14.1 (1.4 to 22.0)	23.0 (−3.0 to 32.5)	0.387

Results are medians (interquartile ranges).

^a^ Other treatments included PT (without injection), injection (without PT), acupuncture, medications, icing, and yoga.

PT, physical therapy.

## Discussion

In this cohort of 29 female patients, there were significant improvements in mHHS, HOS ADL, and iHOT-33 scores at 3–5 years after the baseline visit. Improvements in HOS ADL and iHOT-33 scores were both clinically and statistically significant. Ninety-three percent (*n* = 27) of patients received PT, and 41% (*n* = 12) received injections. Eleven of the 12 patients who received injections also underwent PT. There were no differences in patient-reported outcomes between patients who received both PT and injections versus those who only received PT, injections, or other treatments.

The vast majority of literature focusing on outcomes in women with hip pain have focused on surgical interventions, with women tending to report worse postsurgical outcomes than men. Malviya et al. found significantly lower quality of life scores both before and after hip arthroscopy (mean follow-up: 3.2 years) in women with FAI.^11^ Several other studies have also shown that women report worse functional outcomes before and after hip arthroscopy in comparison with men.^17,18^ In addition, adolescent women are more likely to undergo a second arthroscopic procedure when followed up to 5 years after the initial intervention.^18^ Finally, female sex is a predictor of a longer-than-average recovery time after hip arthroscopy.^19^ However, our results demonstrate significant improvements in pain and function after nonoperative treatments for women with prearthritic hip pain.

It has been established in the literature that females present with greater acetabular anteversion, acetabular inclination, femoral neck anteversion, and decreased lateral center edge angle.^9,20,21^ Nakahara et al. found gender differences in joint orientation and shape around the joint, including the acetabular rim and the femoral neck, ultimately leading to differences in range of motion until bony impingement.^20^ Decreased center edge angle and increased acetabular inclination angles in females suggest a tendency toward dysplasia. Furthermore, Beaule et al. and Ganz et al. observed an increased anterior over-coverage of the acetabular rim in females, suggesting that pincer-type FAI is more common in females.^22,23^ Based on the poor surgical outcomes and anatomical differences in women presenting with hip pain, a further analysis of nonoperative treatments for prearthritic hip and pelvic pain is essential.

Predicated by the poor surgical outcomes in women, nonoperative treatments should be the first step in treating prearthritic or extra-articular hip pain, taking into account all anatomical features and sex differences in the presenting pathology. Our results demonstrate that nonoperative treatments, including targeted PT and injections such as corticosteroids and PRP, were associated with improved patient-reported outcomes at the 3- to 5-year mark after the baseline visit. Based on our results, future studies should investigate higher-powered studies observing the direct relationship between improvements in patient outcomes and nonoperative treatments. By doing so, physicians can gain a better understanding of the efficacy of nonoperative treatments in the prearthritic hip pain population, avoiding unnecessary surgical interventions.

This study is not without limitations. The sample size was relatively small (*n* = 29). However, we believe that our findings provide necessary insight into the long-term outcomes of nonoperative treatments in women with prearthritic or extra-articular hip pain, which are lacking in the literature. As this was a retrospective study with prospective follow-up, we did not have a control group for comparisons. In addition, most patients were between the ages of 43 and 53 years, and were predominantly of Caucasian race, thus limiting the generalizability of our results.

In addition, specific data on nonoperative treatments, such as specific PT exercises and duration of PT, were not compared between participants. Patients were asked to provide as much information as possible regarding treatments they had received during the follow-up period; however, some treatments may have been missed. Finally, this study investigated a cohort of patients who received treatments with prearthritic or extra-articular hip pathologies to gain information on overall long-term outcomes after these treatments; however, future studies should focus on outcomes after specific treatments for specific hip pathologies.

In this cohort of female patients with prearthritic or extra-articular hip pain, nonoperative treatments, such as PT and injections, were associated with improved function and disability outcomes at 3–5 years postbaseline. Future higher-powered studies should investigate the direct relationship between conservative treatments and patient-reported outcomes in women with prearthritic or extra-articular hip pain. Furthermore, more targeted studies should investigate sex-related differences in patient-reported outcomes based on the conservative treatments received, ultimately determining which treatment options work best for specific patient groupings.

## Abbreviations Used

ADL: activities of daily living
FAI: femoroacetabular impingement
HOS: Hip Outcome Score
iHOT-33: International Hip Outcome Tool-33
IQRs: interquartile ranges
MCIDs: minimal clinically important differences
mHHS: modified Harris Hip Score
OA: osteoarthritis
PRP: platelet-rich plasma
PT: physical therapy

## Appendix

**Appendix Table A1.** Physical Therapy Prescription Details

Frequency of therapy	1–2 per week
Duration of therapy	6 weeks
Body locations	Hip and lumbar spine
Reason for referral	Evaluate and treat
Functional activities	Balance training and biomechanics
Modalities	Cold pack and hot pack
Muscle group focus	Hip adductor strengthening, low back strengthening, and glut/hip abductor strengthening
Patient education	Posture and body mechanics
Specific programs	Lumbar spine program and trunk stabilization program
Therapeutic exercise	Home exercise program, active range of motion, passive range of motion, spine stabilization, and core strengthening
Comments	Exercise-based therapy. Core and small muscle control. Stepwise progression with functional goals
